# Aortic Aneurysm Presenting As Deglutition Syncope

**DOI:** 10.7759/cureus.39196

**Published:** 2023-05-18

**Authors:** Fahmi Shibli, Yeseong Kim, Ronnie Fass

**Affiliations:** 1 Gastroenterology, Emek Medical Center, Afula, ISR; 2 Gastroenterology and Hepatology, Temple University Hospital, Philadelphia, USA; 3 Gastroenterology and Hepatology, MetroHealth Medical Center, Cleveland, USA

**Keywords:** aortic aneurysm, syncope, vasovagal reflex, barium esophagram, deglututive syncope

## Abstract

Deglutitive syncope is defined as a neurally mediated syncope in which loss of consciousness occurs during or immediately after swallowing. The causes of deglutitive syncope vary widely and range from intraluminal causes, as well as extra-esophageal compression. In this case report, we present a rare case of deglutitive syncope caused by a thoracic aortic aneurysm compressing the proximal esophagus, a clinical entity described in the literature as dysphagia aortica.

## Introduction

Deglutitive syncope, also known as swallow syncope or syncopal dysphagia, is a rare cause of lightheadedness or loss of consciousness occurring during or immediately after swallowing [1]. The underlying mechanism of this type of syncope is an abnormal stimulation of vagal afferents from the esophagus during deglutition [2], causing atrio-ventricular nodal block, bradycardia, and even sinus arrest, ultimately resulting in cerebral hypoperfusion [3]. Tensoreceptors are activated by the passage of intraluminal bolus in the presence of an intraluminal anatomical aberrancy such as stricture, ring, neoplasm, or external compression of the esophagus; this leads to esophageal distension and the abnormal stimulus of vaso-vagal reflex mediators and symptoms [4].

## Case presentation

An 86-year-old male with a past medical history significant for coronary artery disease, status post coronary artery bypass graft (CABG) 18 years prior to his current presentation with re-do 15 years later, congestive heart failure with reduced ejection fraction, persistent atrial fibrillation, hypertension, gastroesophageal reflux disease, benign prostatic hyperplasia, and status post transurethral resection of the prostate visited our esophageal clinic for evaluation of dysphagia and syncope episodes. The patient reported esophageal dysphagia soon after his CABG re-do. At the time, he noted to have several episodes of dysphagia for solids and pills but not liquids. His dysphagia worsened after suffering several transient ischemic attacks and a pontine ischemic stroke four months prior. Subsequently, a percutaneous endoscopic gastrostomy tube was placed due to the patient’s dysphagia and pulmonary aspirations. However, the patient continued to consume both solids and liquids orally. Consequently, the patient continued to experience almost daily syncopal episodes during oral intake, which resulted in the avoidance of socializing and the need to use a safety helmet during meals. For his deglutitive syncope, the patient underwent an extensive workup in a tertiary referral center including a modified barium swallow with speech pathology evaluation, an upper endoscopy, ENT with laryngoscopy, and high-resolution esophageal manometry which were all normal.

**Figure 1:**
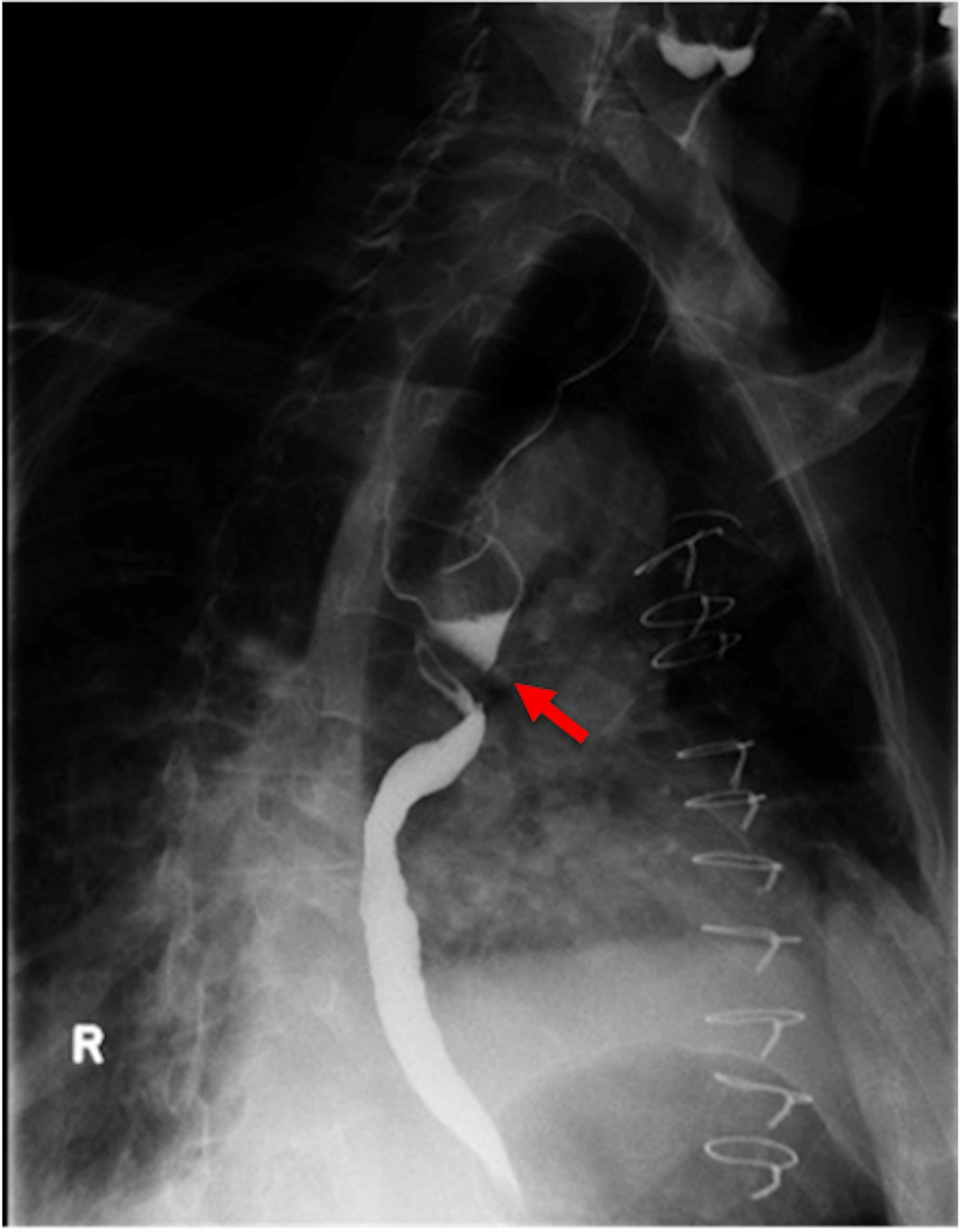
Barium esophagogram shows dilatation of the proximal third of the esophagus above the level of the left mainstem bronchus (see arrow). The esophagus demonstrates a normal caliber distal to the area of extrinsic compression. The area of apparent esophageal narrowing is due to compression between the left mainstem bronchus anteriorly and the tortuous thoracic aorta aneurism posteriorly

When the patient was seen in the esophageal clinic, he reported weight loss secondary to his dysphagia. The patient denied hoarseness, sore throat, hemoptysis, or purulent cough. Furthermore, he stated that he had four syncopal episodes during mealtimes for the last four months, and all were associated with choking and increased coughing spells with no prodromal symptoms. The patient was not receiving an anti-hypertensive at the time. The patient was first referred to a cardiologist for evaluation of possible cardiac arrhythmia as the underlying cause for the deglutitive syncope. Holter ECG for 24 hours monitor revealed atrio-ventricular block (first degree) following swallowing, resulting in syncope. The arrhythmia was only triggered by swallowing and resolved afterward. Consequently, a single-chamber pacemaker was placed. Due to the patient's negative GI workup and esophageal dysphagia symptoms, a barium esophagogram was ordered. The barium swallow demonstrated dilation of the proximal third of the esophagus due to extrinsic compression, while the distal two-thirds of the esophagus appeared normal (Figure 1).

**Figure 2:**
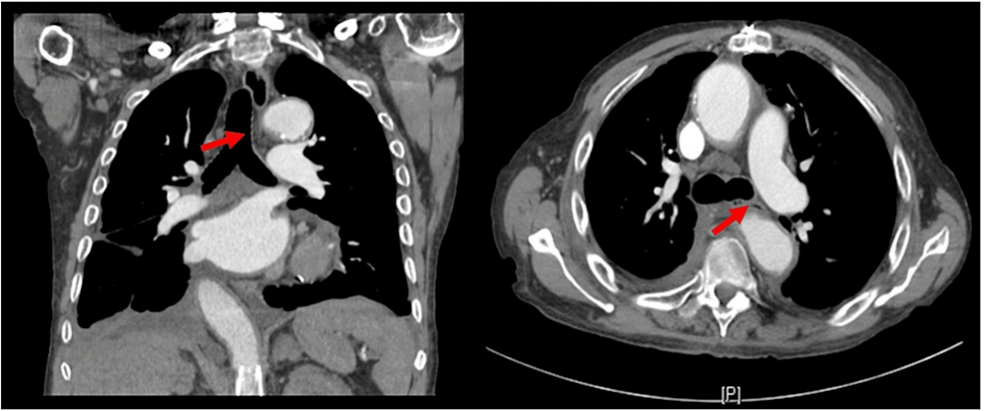
CT chest with and without contrast showed narrowing of the esophagus (see arrow) proximal to the carina due to extrinsic compression between the left main bronchus and the aneurysmal descending thoracic aorta

Concerns about lung mass, enlarged lymph nodes, and thoracic aortic aneurysm were raised. A follow-up chest CT revealed a dilation of the esophagus proximal to the carina due to extrinsic compression from a 7 cm aneurysm of the descending thoracic aorta (Figure 2).

Due to the patient’s frailty and comorbidities, he was not a candidate for surgical aortic aneurysm repair. However, after the placement of the pacemaker, the patient reported complete resolution of the deglutitive syncope episodes that he has experienced. Eventually, the patient succumbed to his comorbidities and died from a systemic infection.

## Discussion

Since being first described in 1793, deglutitive syncope has been sporadically reported in the literature, mostly in case reports [5]. Approximately 40% of the cases reported between 1793 and 2011 were attributed to an underlying digestive disorder [6]. The majority of syncopal episodes can be explained by an underlying cardiovascular disease causing cardiac arrhythmia or abnormal response to vaso-vagal stimulation. Deglutitive syncope represents a subset of disorders causing neurally mediated syncope. The mechanism behind deglutitive syncope involves inappropriate activation of the vagal nerve fibers which innervate both the esophagus and the heart, resulting in atrio-ventricular block, bradycardia, or even short asystole leading to a sudden reduction in cardiac output which can result in syncope [7]. The stimulus for generating vagal impulses varies widely and includes bolus transit that causes mechanical distention of the esophageal wall which could be excessive in various conditions such as esophageal stricture and external compression and was even described in patients with achalasia [8-10]. In our patient, the vaso-vagal stimulation resulted in documented cardiac arrhythmia that was documented with ECG holder monitoring during dysphagia spells.

The initial workup of deglutitive syncope involves a comprehensive evaluation for common causes of syncope and includes an electrocardiogram, echocardiogram, Holter monitor, tilt-table test, carotid artery Doppler ultrasound, brain imaging, exercise stress test, and cardiac catheterization. Cessation of the syncopal episodes should be pursued to prevent serious injury to the patient. Thus, even prior to the identification of the underlying cause, intervention to avert the cardiac arrhythmia that leads to the syncopal episode, such as pacemaker placement, should be considered [11]. From a GI point of view, the initial set of studies to evaluate the cause of deglutitive syncope includes upper endoscopy, esophageal manometry, pH impedance, modified barium esophagogram, laryngoscopy, and speech pathology evaluation. These studies, however, mainly focus on intraluminal causes of deglutitive syncope. Some of these etiologies include motility disorders, esophageal strictures, esophageal carcinoma, and mechanical distension via food bolus. Imaging studies that include a barium swallow or CT of the chest can be useful in identifying the extraluminal causes of deglutitive syncope, which include hiatal hernia, intrathoracic mass, or vascular malformations. The diagnosis was made when the tests performed to rule out intraluminal causes were negative, and a CT clearly showed external compression of the esophagus due to the aortic aneurysm, rendering deglutitive syncope the most likely cause.

Treatment of deglutitive syncope is directed toward managing the underlying cause. Trigger foods should be avoided such as cold/hot foods, carbonated beverages, or certain pills (4). In cases where an identifiable esophageal pathology, such as an esophageal stricture or esophageal spasms, is associated with symptoms, medical or surgical management may be curative [12]. If surgical intervention is not feasible, as is the case with this patient, pacemaker implantation and lifestyle modifications related to type and amount of food consumption are considered [13-14]. Cardio-inhibitory medications, such as beta-blockers or calcium channel blockers, should be discontinued if possible, and anticholinergics or sympathomimetics, such as atropine or dobutamine, which downregulate vagal activation and increase cardiac output, may be added [15].

This case is unique because it demonstrated that dysphagia aortica was the cause of deglutitive syncope. Dysphagia aortica in itself is an uncommon cause of dysphagia. Dysphagia aortica develops due to a large, atherosclerotic, tortuous, or aneurysmal aorta which causes extrinsic compression or impingement on the esophagus [16]. There is a paucity of cases reporting that deglutitive syncope is caused by aortic malformations [17] or post-surgical manipulation of the aorta [18]. Our case highlights the importance of considering extrinsic compression of the esophagus as a cause for deglutitive syncope or any unexplained dysphagia. Furthermore, workup that included modified barium swallow, laryngoscopy, speech pathology, upper endoscopy, and high-resolution esophageal manometry was unable to identify the extrinsic compression because of the high location of the latter in the esophagus. Regardless, diagnosis of the aortic aneurysm as the cause of patients’ deglutitive syncope requires high suspicion and the need for a barium swallow and CT of the chest. Furthermore, treatment requires addressing the underlying mechanism. Any type of esophageal dilatation is unlikely to help.

## Conclusions

We describe here a rare case of a patient with an aortic aneurysm creating an external pressure on the proximal esophagus which resulted in vaso-vagal-mediated deglutition syncopal episodes. Deglutitive syncope, although rare, should remain on the differential when evaluating patients who present with syncope, especially when a clear temporal association with mealtimes is present. Tests and images such as EGD, manometry, and CT scans to evaluate for intraluminal as well as extra-luminal sources must be obtained as part of the workup. When present, an aortic aneurysm can cause extrinsic compression of the esophagus, causing an entity also known as dysphagia aortica, which leads to syncopal episodes with swallows. When identified, surgical fixation is the preferred modality of treatment. If this is not an option, treatment of the ensuing atrio-ventricular block via pacemaker implantation is also an effective management modality.
